# Frederick the Noble
*"Frederick the Noble." By Sir Morell Mackenzie. London: Sampson Low, Marston, Searle, and Rivington. Price 2*s*. 6*d*.


**Published:** 1888-10-20

**Authors:** 


### Frederick the Noble.*

Undoubtedly Sir Morell Mackenzie's "Frederick the Noble" is the book of the hour. In all Europe there cannot be many who do not share in a curiosity, not wholly vain or irreverent, with regard to the last days of a monarch who, though his career had been stainless and honourable, showed himself more than ever a hero and a king under the shadow of death. That the peace and sacredness that should have surrounded his deathbed were marred by jealousy and backbitings among his doctors is infinitely to be regretted, but it cannot be denied that these unseemly accusations have aroused public interest in the purely technical aspects of the case to an exceptional degree. Sir Morell Mackenzie's statement is the full, complete, and authoritative on the illness of the Emperor Frederick and the circumstances that attended it; and while it was inevitable that the writer should devote a considerable amount of his space to refuting the charges brought against him, the minute account he gives of the progress of the case is the best proof of the care and honesty he displayed in attending his royal patient. It is, however, to be regretted that we have not received the "official case" as it is called with the P.M. reports which were published by the German Government about six months ago, and to which this book is in part a reply. We cannot commend Sir Morell Mackenzie for preventing the sale in England of the English translation of the official case, if the statement that he has done so prove correct. Without the two sides it is neither possible nor reasonable to attempt to form a just judgment on the whole case.

It seems strange, indeed all but incredible, that the most irresponsible of journalists should have tried to depreciate a doctor's reputation by representing him as of Jewish descent in order to rouse the strong anti-Semitic prejudice which marks so many Continental nations. The reasoning on this point was certainly original, the chief point being that Morell Mackenzie was the "English equvalent" of "Moritz Marcovicz," which it was said was Sir Morell's real appellation. It is a pity that the writer did not know that "Morell Mackenzie" is an obviously Scotch name, for then he might have relied for evidence on the legend that the lost ten tribes of Israel settled in Aberdeen; or invented a myth of his own to the effect that Scota, that daughter of Pharaoh who protected Moses, might, when she fled with her husband Gathelus to settle on, and give her name to, the Caledonian shore, have taken a few favoured Hebrews in her train. On the whole, the journalist who invented the Judaic fable did his lying pretty badly.

There can be no doubt that an attempt was made to harass the English surgeon, and to put him apparently in the wrong. He himself says: "Two things supported me in what otherwise would have been an intolerable position. First, my own consciousness of perfect integrity of purpose; secondly, the absolute trust and delicate consideration with which from first to last I was treated by my noble-hearted patient. No physician could wish for a patient more obedient to his injunctions, more full of 'sweet reasonableness' than the ruler of the mighty Empire of Germany." To this statement he adds the remark:—"I think I have also some grounds of complaint against the Prussian Government, which, whilst allowing my adversary free access to the State archives, refused me the same privilege. As I have shown in the body of this little work, these official sources are of a very miscellaneous character, but among them there are important documents relating to the case of the late Emperor which, in justice to me, should not have been kept from the public. Amongst others may be mentioned protocols of Professor Von Schrotter, Dr. Krause, and myself, drawn up in November, 1887, and, more especially, the written refusal of the Emperor (then Crown Prince) to submit to any other external operation than tracheotomy. The protocols which it is proved that Professors Gerhardt and Von Bergmann sent in to the Hans Ministerium would also furnish interesting reading, and would show what really were the views of these gentlemen before I was summoned to Berlin. The first report of Professor Virchow would also be highly instructive. I can only hope that these documents and other matters bearing on this historical case will some day be made public. I at least have no reason to fear the full light of day."

The Emperor's refusal to submit to any operation more severe than tracheotomy is a matter of great importance as silencing those people, wise after the event, who insist that complete extirpation of the larynx should have been attempted. Sir Morell Mackenzie quotes the statement of an eminent German authority that in this operation—technically called thyrotomy—it is a triumph for the operator if the patient does not actually die under the knife; and the statistics given concerning it are such as to justify the Emperor and Empress in shrinking from such a worse than forlorn hope. "With the palliative treatment (including tracheotomy) life is preserved under almost normal conditions for at least one year, and in a less favourable state for at least one year longer—altogether, for two years. With the radical treatment (the thyrotomy) life is sacrificed at once, as the result of the operation is 27·2 per cent. of cases, whilst in over 54·54 per cent. death is hastened owing to the greater activity of the morbid process set up by the operation, and in these cases the condition of existence is rendered less favourable by the premature use of a tracheotomy tube, necessitated by unsuccessful thyrotomy. A complete cure has been twice obtained, or 9·09 per cent. These two are the only successful cases which are known to have followed the operation of thyrotomy. Of thirty-five operations for partial extirpation of the larynx, fifteen proved fatal, or 42·85 per cent. In regard to total extirpation of the larynx, this operation, when not immediately fatal, leaves the patient in abject misery."

It is hardly necessary to quote the account of how the Crown Prince received the announcement made to him at Villa Zirio, that the growth in his throat had developed the symptoms of cancer. Everyone knows that anecdote of simple heroism, but still it may be well to give it in Sir Morell Mackenzie's own words: "Without rising from my chair, I informed his Imperial Highness that a very unfavourable change had taken place in his throat. He said, 'Is it cancer?' to which I replied, 'I am sorry to say, sir, it looks very much like it, but it is impossible to be certain.' I felt that evasive answers—which, for the patients' own sake, medical men are often compelled to give under similar circumstances—would in the present instance have been out of place. The Crown Prince received the communication with perfect calmness. After a moment of silence, he grasped my hand and said, with that smile of peculiar sweetness which so well expressed the mingled gentleness and strength of his character, 'I have lately been fearing something of this sort. I thank you, Sir Morell, for being so frank with me.' In all my long experience I have never seen a man bear himself under similar circumstances with such unaffected heroism. He showed not the least signs of depression, but spent the day in his ordinary occupation, and at dinner time that evening he was cheerful, without apparent effort, and chatted freely in his usual manner."

Although it is evident from his statements that the English physician suffered considerable annoyance from

* "Frederick the Noble." By Sir Morell Mackenzie. London: Sampson Low, Marston, Searle, and Rivington. Price 2s. 6d.

the jealousy of some of his German colleagues, and also that he thought these gentlemen in many respects incompetent, he speaks courteously and dispassionately of all except Professor Von Bergmann, and even admits that a detail in Dr. Bramann's way of performing tracheotomy—the holding the wound open till the bleeding ceased before inserting the canula—is an improvement on the English method. But with Von Bergmann he is naturally indignant. This doctor not only established a regular system of espionage over Sir Morell's actions, but interfered with him in a fashion so brutal and injurious to the Emperor, that the latter afterwards alluded to the 12th of April—"the fatal 12th of April," Sir Morell calls it, as "the day when Bergmann ill-treated me." This is the account given of the occurrence: "As the tube which I now proposed to try was different in shape from any of those which had been used since the case had been formally given up to me by Professor Von Bergmann at San Remo, I thought that professional courtesy required that I should ask him to be present on the occasion. As I intended to do whatever was necessary with my own hands, there was really no need for the assistance of a surgeon; but it is an elementary rule of civilised medical practice that all those associated together in the management of a case should be made acquainted with the details of the treatment that is carried out. As soon as the new tube was ready, therefore, I despatched a messenger to Professor Von Bergmann to request him to come to me as soon as possible, meaning, of course, that I was anxious to proceed to change the tube without delay. In sending off that message, little did I think that it would have such fatal consequences. It is no exaggeration to say that these hastily-scribbled lines proved to be the death-warrant of the Emperor. Had I had the slightest idea of what was to follow I should certainly not have allowed any over-punctilious notions of etiquette to mislead me into taking so disastrous a step. At the moment, however, it appeared to be the right thing to do. . . . We then proceeded to the Emperor's room, accompanied by Mr. Hovell, each of us carrying several tubes. We found the Emperor engaged in writing. The inspiration was distinctly audible, but beyond this there was not the slightest indication of any difficulty in breathing. Professor Von Bergmann placed a chair opposite the window, and asked the Emperor to sit down upon it, and thereupon, without making any remark, he quickly undid the tape which kept the canula in position, pulled the latter out, and with considerable force endeavoured to insert one which he had in his hand, and which was not provided with a pilot. The instrument was forced into the neck, but no air came through it. The Emperor's breathing thereupon became very much embarassed, and the Professor withdrew the tube. This was followed by a violent fit of coughing, and there was considerable hæmorrhage. Professor Von Bergmann next seized a tamponcanula covered with sponge, cut the sponge quickly off, and tried to push the tube into the windpipe. Again, no air came through the canula, and it was clear that, instead of entering the air passage, it had been forced downwards in front of the trachea, ploughing up the soft tissues in that situation, and making what is technically known as a 'false passage.' Again the professor had to pull out the tube, and again its withdrawal was followed by violent coughing and streams of blood. To my dismay, Professor Von Bergmann then pushed his finger deeply into the wound, and on withdrawing it tried to insert another tube. He again failed, however, and again the attempt was followed, as before, by most distressing coughing and copious bleeding. Professor Von Bergmann then asked that his assistant, who was waiting in his carriage outside, might be sent for. It seemed as if he contemplated doing some further operation, perhaps enlarging the wound, but the Emperor was saved any further torture by the arrival of Dr. Bramann on the scene. Professor Von Bergmann at once yielded the case into the hands of his assistant, and the young surgeon, taking a moderate sized canula (No. 8 German measure), passed it with the greatest ease into the trachea."

Professor Gerhardt also comes in for very severe blame for having used the galvano-cautery with such barbarous frequency in the early stages of the disease. The Crown Prince's throat had been burned every day for nearly a fortnight, the usual frequency with which this operation is performed being once, or at most twice, a-week. Sir Morell protests that he knew not which to be most astonished at—the therapeutic energy of the physicians or the endurance of the patient.

There are many allusions, reverent and well-deserved, to this quiet, manly endurance, when strength and patience must have been strained to the utmost, and the Royal sufferer must have been tried with doubts as to the utility of his physicians' efforts. Even to the last he never failed in thought and consideration for those who attended on him; and while time will test the value of Sir Morell Mackenzie's book in many ways, it may safely be predicted that nothing will come that will give cause for any change in the title he gives his patient—Frederick the Noble.

